# Efficacy of Prophylactic Negative-Pressure Wound Therapy with Delayed Primary Closure for Contaminated Abdominal Wounds

**DOI:** 10.1155/2022/6767570

**Published:** 2022-11-14

**Authors:** Yo Sato, Eiji Sunami, Kenichiro Hirano, Motoko Takahashi, Shin-ichi Kosugi

**Affiliations:** ^1^Department of Digestive and General Surgery, Uonuma Kikan Hospital, 4132 Urasa, Minami-Uonuma, Niigata 9497302, Japan; ^2^Uonuma Institute of Community Medicine, Niigata University Medical and Dental Hospital, 4132 Urasa, Minami-Uonuma, Niigata 9497302, Japan

## Abstract

**Background:**

Prophylactic negative-pressure wound therapy (NPWT) to prevent surgical site infection (SSI) may be effective for severely contaminated wounds. We investigated the safety and efficacy of NPWT with delayed primary closure (DPC) for preventing SSI.

**Methods:**

For patients with contaminated and dirty/infected surgical wounds after an emergency laparotomy, the abdominal fascia was closed with antibacterial absorbent threads and the skin was left open. Negative pressure (−80 mmHg) was applied through the polyurethane foam, which was replaced on postoperative days 3 and 7. DPC was performed when sufficient granulation was observed. The duration and adverse events of NPWT, the development of SSI, and the postoperative hospital stay were retrospectively reviewed.

**Results:**

We analyzed the cases of patients with contaminated (*n* = 15) and dirty/infected wounds (*n* = 7). The median duration of NPWT was 7 days (range 5–11 days). NPWT was discontinued in one (4.5%) patient due to wound traction pain. SSI developed in seven patients (31.8%), with incisional SSI in one (4.5%) and organ/space SSI in six (27.3%). The median postoperative hospital stay was 17 days (range 7–91 days). There was no significant relationship between postoperative hospital stay and wound classification (*P*=0.17) or type of SSI (*P*=0.07).

**Conclusion:**

Prophylactic NPWT with DPC was feasible and may be particularly suitable for severely contaminated wounds, with a low incidence of incisional SSI.

## 1. Introduction

Surgical site infection (SSI) frequently occurs in contaminated wounds after abdominal surgery, resulting in frequent dressing changes and poor appearance after healing. SSI is a risk factor for incisional hernia, which may impair the patient's quality of life [1]. A prolonged hospital stay due to SSI also increases medical costs [2]. All of these are medical and socioeconomic problems that should be avoided.

Various approaches to suppress SSI have been taken, including the use of a subcutaneous drain [3], delayed primary closure (DPC) [4], and SSI bundle [5]; however, these methods have not sufficiently decreased the rate of SSI. Negative-pressure wound therapy (NPWT) has attracted attention recently; its use promotes wound healing by inducing angiogenesis, proliferating fibroblasts, and increasing granulation tissue [6, 7]. NPWT was initially used to treat chronic wounds and tissue defects [8, 9], but it is now used to prevent SSI even in abdominal surgical wounds.

Two NPWT methods have been widely used as prophylaxis for SSI after abdominal surgery: closed NPWT and open NPWT. In closed NPWT, the wound is primarily closed, and a commercially available, simple, and portable device is applied on the wound surface to indirectly suck the closed wound. In open NPWT, only the fascia is closed, and the skin and subcutaneous tissue are left open; a porous filler is adjusted to the wound size by hand and applied directly to suck the open wound. Open NPWT is considered to have an advantage in that granulation growth may be accelerated by using the filler directly [7]. However, a disadvantage of open NPWT is that the time to wound closure is extended [10]. In this condition, DPC is used after open NPWT to shorten the time to wound closure [11–13].

Although several randomized controlled trials have evaluated the use of closed NPWT in patients who underwent elective surgery with less intraabdominal contamination, the efficacy and safety of NPWT have not been determined because the preventive effect of SSI varied among trials and studies even in a meta-analysis [14–18]. On the other hand, a low incidence of SSI was reported with the use of NPWT for contaminated wounds [11–13, 19–21]; NPWT may thus be suitable for use on severely contaminated wounds. We conducted the present study to examine the safety and efficacy of open NPWT with DPC for contaminated wounds.

## 2. Patients and Methods

### 2.1. Patients

A total of 239 patients underwent open abdominal surgery at Uonuma Kikan Hospital between April 2016 and May 2018. Of these, we analyzed the cases of the 22 (9.2%) patients who had class III (contaminated) or IV (dirty/infected) wounds according to the U.S. Centers for Disease Control and Prevention (CDC) classification and received NPWT. We entered the cases into a prospective database. This study was approved by the Ethics Committee of Uonuma Kikan Hospital, which waived the requirement for patient consent due to the study's retrospective design (No. 2021-3-001). All procedures followed were in accordance with the ethical standards of the hospital and national committees on human experimentation and with the Helsinki Declaration of 1964 and later versions.

### 2.2. Treatment Protocol

In all 22 patients, the abdominal fascia was closed with interrupted suture using 0-coated polyglactin 910 with triclosan (VICRYL PLUS™, Ethicon, Johnson & Johnson, Somerville, NJ, USA) and/or running suture using 0-polydioxanone (PDS II™, Ethicon, Johnson & Johnson), with the wound left open. The subcutaneous defect of the wound was filled with polyurethane foam, and negative pressure (−80 mmHg) was applied with a RENASYS™ negative-pressure wound therapy system (Smith & Nephew, Watford, UK). A single surgeon (YS) changed the dressing and examined the status of each wound routinely on postoperative days (POD) 3 and 7.

Wound closure was defined based on the observation of healthy granulation tissue on the exposed fascia with no clinical signs of infection on POD 7. Delayed suture using 3-0 nylon monofilament (Keisei Medical Industrial, Tokyo) or wound taping was performed as appropriate. NPWT was continued another 4 days if the granulation on the exposed fascia was not sufficient. All patients received scheduled follow-up within 30 days after surgery.

### 2.3. Evaluation

The development of SSI was evaluated using the criteria for defining a surgical site infection, as stated in the Guideline for the Prevention of Surgical Site Infection [22]. We obtained the patient characteristics, causes, and the degree of intraabdominal contamination according to the upper (stomach and duodenum) or lower (small and large bowel) gastrointestinal diseases, the duration of NPWT application, the occurrence of adverse events caused by NPWT, delayed wound closure methods, and the length of postoperative hospital stay from prospectively collected data.

### 2.4. Statistical Analysis

Continuous variables are presented as medians and ranges because of non-normal distributions and were compared using the Mann–Whitney *U* test or Kruskal–Wallis test. Categorical variables are presented as numbers and percentages and were compared using the *χ*^2^-test or Fisher's exact test. A two-tailed*P* value <0.05 was considered significant. All statistical analyses were conducted using SAS JMP ver. 14.0.1.

## 3. Results

### 3.1. Patient Characteristics

The patient characteristics are summarized in Table 1. All operations were performed in an emergency setting. The causes of intraabdominal contamination and the CDC wound classifications are listed in Table 2. Operative wounds were contaminated due to upper and lower gastrointestinal diseases in 5 and 17 patients, respectively. Six patients had an intraoperative small bowel injury, and one had a traumatic injury due to a traffic accident. There were two patients with perforated colon cancer and one with perforated malignant lymphoma of the ileum. Other causes of intraabdominal contamination included pancreatic necrosis after endoscopic sphincterotomy, peristomal abscess, intraabdominal abscess after appendectomy, and idiopathic perforation of the ileum (one patient each).

### 3.2. Outcomes of Prophylactic NPWT

NPWT was in place for a median of 7 days (range 5–11 days). Three patients required NPWT for 11 days. NPWT was discontinued 5 days after application in one patient with postoperative delirium who did not tolerate NPWT and another patient with NPWT-associated pain. Eleven and eight patients received delayed suture and wound taping, respectively. The remaining three patients required no adaptation procedure because their wounds were likely to close spontaneously.

Surgical site infection (SSI) developed in seven (31.8%) patients, including one (4.5%) superficial/deep incisional SSI and six (27.3%) organ/space SSIs (Table 3). There was no significant relationship between the CDC wound classification and incisional SSI (*P*=1.00) or organ/space SSI (*P*=0.61). The median postoperative hospital stay was 17 days (range 7–91 days), and it had no significant relationship with the CDC wound classification (*P*=0.80) or the delayed wound closure method (*P*=0.74) (Table 4). Although not significant, the patients with organ/space SSI tended to have longer postoperative hospital stays than those with a superficial/deep incisional SSI or no SSI (*P*=0.07).

## 4. Discussion

Our present findings demonstrated that the prophylactic use of open NPWT with DPC for contaminated wounds after open abdominal surgery was safe and feasible, as NPWT-associated adverse events and incisional SSI occurred in only one patient each (4.5%) in this study. All patients completed our fixed protocol within 11 days, and the median postoperative hospital stay was 15 days. Several studies have examined closed NPWT for noncontaminated wounds [14–18], but open NPWT with DPC for only contaminated wounds has scarcely been investigated.

The incidence of incisional SSI when only primary closure was performed on contaminated wounds was ≥50% [13], and even DPC provided no significant reduction of incisional SSI (against expectations) [14]. On the other hand, Glass et al. reported that open NPWT using a filler promotes granulation growth and exerts a bacteriostatic effect on the wound surface, suppressing nonfermentative Gram-negative bacilli including *Pseudomonas* species in particular [23]. It was reported that the use of NPWT with DPC reduced the incidence of incisional SSI from 63.2% to 10.7% [13] and from 37% to 0% [12]. The incidence of incisional SSI in the present study was 4.5%, which was comparable to these earlier reports. Notable, no incisional SSI was observed in the present cases with a class IV wound, which is regarded as the highest degree of contamination.

It is well known that the development of an SSI prolongs the length of hospital stay and increases medical costs [24]. Our present findings indicate that the use of open NPWT with DPC for contaminated wounds may lead to shorter hospital stays and lower medical costs.

The NPWT was completed within the median of 7 days, and the median length of hospital stay was 17 days in the present series. The length of hospital stay might vary widely among patients due to the differences in the primary disease, the patients' general condition at the time of surgery, and the occurrence of organ/space SSI or other complications; for example, the present patients with organ/space SSI were likely to have longer postoperative hospital stays compared to those without this type of SSI (*P*=0.07). In the previous investigations of open NPWT with DPC, mainly the incidence of incisional SSI was reported, not the length of hospital stay. A meta-analysis focusing on closed NPWT used in elective surgery showed that the length of hospital stay was 0.47 days shorter than that following conventional wound dressing (95% confidence interval, −0.71 to −0.23; *P* < 0.0001) [25]. A subgroup analysis of that study showed a greater shortening effect of −5.1 days in colorectal surgery, which is regarded as posing a higher degree of wound contamination than other surgeries [25]. NPWT might have a greater effect for shortening the length of hospital stay in proportion to the degree of wound contamination.

Few studies have compared open and closed NPWT for contaminated wounds. Frazee et al. reported that the incidence of incisional SSI was 4.2% (1/24) in open NPWT and 8% (2/25) in closed NPWT (*P*=1.0) [10]. Because of that study's limited sample size, these data should be assessed by further investigations with larger sample sizes. In the Frazee et al. report, DPC was not performed after open NPWT (with the median time to wound healing of 48 days), whereas the corresponding time in closed NPWT was 7 days (*P* < 0.0001) [10]. In our present study, DPC was added for all patients, and the median time to closure was 7 days, with a comparable incisional SSI rate. Based on these results, it seems desirable to add DPC to open NPWT.

Open NPWT has the advantage of enabling sequential evaluations of the condition of granulation tissue and the presence of necrotic tissue in the wound bed, where additional debridement can also be performed appropriately, especially in wounds in poorer condition such as contaminated wounds. Moreover, adding DPC to open NPWT might shorten the time to wound closure, as is observed for closed NPWT.

The limitations of this study are as follows: (1) a small number of patients were analyzed because this was a single-institution study conducted only for patients with contaminated wounds after open abdominal surgery. (2) Our hospital was newly established in June 2015, so we did not have enough historical data to compare. (3) The cost-effectiveness of NPWT was not analyzed because of the study's single-arm, noncomparative design. (4) It was uncertain whether our 7-day protocol was optimal, although angiogenesis and the growth of granulation tissue during NPWT are reported to progress within 3–10 days [6]. Ma et al. also reported that NPWT significantly improved angiogenesis that preceded granulation in dermal regeneration from days 3 to 7 compared to gauze dressing [6]. These findings were consistent with our clinical observations; it thus seemed rational to continue NPWT for at least 7 days. (5) It was also uncertain whether the negative pressure at −80 mmHg was optimal, although we selected the recommended negative pressure according to the NPWT system manufacturer's instructions. In their review, Birke et al. recommended the negative pressure values within −50 to −150 mmHg considering the tissue blood flow, granulation growth, wound contraction, and microdeformation, and they also recommended setting a lower negative pressure value to improve pain [26]. It seems acceptable that only one patient had traction pain in the present study.

In conclusion, we performed open NPWT with DPC for contaminated and dirty/infected surgical wounds and were able to safely treat these wounds. This method can be applied flexibly for wounds of differing status, and it can lower the incidence of incisional SSI. The method is particularly suitable for severely contaminated wounds. Further investigation comparing this method with closed NPWT in terms of cost-effectiveness and optimal treatment indications is warranted.

## Figures and Tables

**Table 1:** Patient characteristics.

	Number (%)
Female	14 (64)
Age	76 (44–92)^*∗*^
Body mass index	22.9 (15.8–29.3)^*∗*^
Diabetes mellitus	6 (27)
Corticosteroid usage	2 (9.0)
Anticoagulation	6 (27)
Dementia	6 (27)
ASA classification	
2	8 (36)
3	10 (46)
4	4 (18)
Operative time (minutes)	102 (41–344)^*∗*^
Blood loss (mL)	265 (0–1753)^*∗*^
CDC wound classification	
Class III	15 (68)
Class IV	7 (32)

^*∗*^Data are depicted as median (range). ASA: American Society of Anesthesiologists; CDC: Centers for Disease Control and Prevention.

**Table 2:** Causes of intraabdominal contamination and CDC wound classification.

Causes	Number (%)	Class III/IV
Upper gastrointestinal diseases		
Perforated peptic ulcer	2 (9.1)	2/0
Perforated gastric cancer	1 (4.5)	0/1
Post-ESD perforation	1 (4.5)	0/1
Others	1 (4.5)	1/0

Lower gastrointestinal diseases		
Small bowel injury	7 (41.1)	6/1
Perforated diverticulitis	2 (9.0)	1/1
Malignancy	3 (13.6)	1/2
Perforated appendicitis	2 (9.0)	2/0
Others	3 (17.7)	2/1
Total	22 (100)	15/7

CDC: Centers for Disease Control and Prevention; ESD: endoscopic submucosal dissection.

**Table 3:** Surgical site infection and CDC wound classification.

	*n*	Surgical site infection		*P*
		Incisional	Organ/space	
CDC wound classification				0.17
Class III	15	1	5	
Class IV	7	0	1	
Total	22	1 (4.5%)	6 (27.3%)	

CDC: Centers for Disease Control and Prevention.

**Table 4:** The length of postoperative hospital stay according to the wound status.

	*n*	Length of hospital stay (days)^*∗*^	*P*
CDC wound classification			0.80
Class III	15	16 (7–91)	
Class IV	7	18 (9–38)	

Wound closure method			0.74
Delayed primary closure	11	16 (7–91)	
Wound taping	8	20 (9–25)	
None	3	12 (9–23)	

Surgical site infection			0.07
Incisional	1	13	
Organ/space	6	23.5 (18–91)	
None	15	13 (7–38)	

^*∗*^Data are depicted as median (range). CDC: Centers for Disease Control and Prevention.

## Data Availability

The data that support the findings of this study are available from the corresponding author upon reasonable request.

## Abbreviations

CDC: Centers for disease control and prevention
DPC: Delayed primary closure
NPWT: Negative-pressure wound therapy
POD: Postoperative day
SSI: Surgical site infection.
